# Batch Effects during Human Bone Marrow Stromal Cell Propagation Prevail Donor Variation and Culture Duration: Impact on Genotype, Phenotype and Function

**DOI:** 10.3390/cells11060946

**Published:** 2022-03-10

**Authors:** Gabriele Brachtl, Rodolphe Poupardin, Sarah Hochmann, Anna Raninger, Karsten Jürchott, Mathias Streitz, Stephan Schlickeiser, Michaela Oeller, Martin Wolf, Katharina Schallmoser, Hans-Dieter Volk, Sven Geissler, Dirk Strunk

**Affiliations:** 1 Spinal Cord Injury and Tissue Regeneration Center Salzburg (SCI-TReCS), Cell Therapy Institute, Paracelsus Medical University (PMU), 5020 Salzburg, Austria; gabriele.brachtl@pmu.ac.at (G.B.); rodolphe.poupardin@pmu.ac.at (R.P.); sarah.hochmann@pmu.ac.at (S.H.); anna.raninger@pmu.ac.at (A.R.); martin.wolf@pmu.ac.at (M.W.); 2 Center for Regenerative Therapies (BCRT), Berlin Institute of Health (BIH), Charité Universitätsmedizin Berlin, 13353 Berlin, Germany; karsten.juerchott@bih-charite.de (K.J.); mathias.streitz@fli.de (M.S.); stephan.schlickeiser@charite.de (S.S.); hans-dieter.volk@charite.de (H.-D.V.); sven.geissler@charite.de (S.G.); 3 Friedrich-Loeffler-Institut, Federal Research Institute for Animal Health, Insel Riems, 17493 Greifswald, Germany; 4 Department of Transfusion Medicine and SCI-TReCS, Paracelsus Medical University (PMU), 5020 Salzburg, Austria; m.oeller@salk.at (M.O.); k.schallmoser@salk.at (K.S.); 5 Berlin Center for Advanced Therapies (BeCAT), Charité Universitätsmedizin Berlin, 13353 Berlin, Germany; 6 Institute of Medical Immunology, Charité Universitätsmedizin Berlin, 13353 Berlin, Germany

**Keywords:** cell therapy, regenerative medicine, donor variation, batch effect, human platelet lysate (hPL), bone marrow, stem cells, chondrogenesis

## Abstract

Donor variation is a prominent critical issue limiting the applicability of cell-based therapies. We hypothesized that batch effects during propagation of bone marrow stromal cells (BMSCs) in human platelet lysate (hPL), replacing fetal bovine serum (FBS), can affect phenotypic and functional variability. We therefore investigated the impact of donor variation, hPL- vs. FBS-driven propagation and exhaustive proliferation, on BMSC epigenome, transcriptome, phenotype, coagulation risk and osteochondral regenerative function. Notably, propagation in hPL significantly increased BMSC proliferation, created significantly different gene expression trajectories and distinct surface marker signatures, already after just one passage. We confirmed significantly declining proliferative potential in FBS-expanded BMSC after proliferative challenge. Flow cytometry verified the canonical fibroblastic phenotype in culture-expanded BMSCs. We observed limited effects on DNA methylation, preferentially in FBS-driven cultures, irrespective of culture duration. The clotting risk increased over culture time. Moreover, expansion in xenogenic serum resulted in significant loss of function during 3D cartilage disk formation and significantly increased clotting risk. Superior chondrogenic function under hPL-conditions was maintained over culture. The platelet blood group and isoagglutinins had minor impact on BMSC function. These data demonstrate pronounced batch effects on BMSC transcriptome, phenotype and function due to serum factors, partly outcompeting donor variation after just one culture passage.

## 1. Introduction

Important successes in skeletal regenerative medicine must not obscure the fact that a panacea for nonunion fractures has not yet been found. Durable cartilage regeneration also remains elusive [1]. Sophisticated protocols for skeletal stem-cell activation will help in this regard [2]. BMSCs, representing the prototype mesenchymal stromal cells, are a versatile source of cells for skeletal regeneration [3]. However, culture conditions to direct reproducible bone and cartilage differentiation of BMSC are still not fully defined [4].

Organoid-like 3D BMSC differentiation models may provide more realistic readouts for studying osteochondral differentiation [5,6]. We have previously shown that cell culture-expanded BMSCs, but not stromal cells from white adipose tissue or umbilical cord, preserve their potential to create 3D organoid-like cartilage templates in vitro and fully re-establish bone and the hematopoietic marrow niche in vivo, in the presence of human platelet lysate (hPL) [7,8]. BMSC-derived scaffold-free cartilage organoid-like disk transplants could completely regenerate critically sized femur defects in a humanized mouse model. The skeletal regeneration competence was found to be predetermined by a discerning epigenetic landscape enabling common transcription factors to act on genes involved in ossification [9].

Batch effects in omics data are considered to occur when differences in experiment time, handling or reagent lots, to name some, result in a different outcome of the analysis [10]. In cell therapy, donor variation is generally considered to play an important role [11,12,13], but batch effects are barely considered. Pleiotropic raw materials for cell manufacture, such as animal-derived or human serum and undisclosed proprietary components of defined serum-free media, comprise a risk of deviating cell quality and quantity. More importantly, serum lot, source, or other unknown variables, can precipitate batch effects changing gene expression or any other outcome of interest in cell products [14].

Application of hPL as an efficient substitute for fetal bovine serum (FBS) in stromal cell culture has become common practice since the 2000s, following recommendations of the European Medicines Agency to avoid animal-derived raw materials for manufacturing cell therapeutics [15]. Notably, hPL production from so-called buffy-coats, derived from whole-blood donations as a side product of clinical red blood cell unit production, or from platelet-rich plasma collected by single-donor platelet apheresis, is highly divergent among producers [16] and efforts for standardization are still ongoing [17]. Besides the number of pooled donations and the mode of platelet processing influencing hPL quality, ABO blood group antigens of platelets and corresponding isoagglutinins in donor plasma may also be critical for cultured cells [18]. Therefore, we have compared the effect of hPL pooled either from fresh platelet concentrates of blood group O platelets (without AB antigens) and AB plasma (without isoagglutinins, hPL O/AB), with hPL pooled from expired platelet concentrates of mixed ABO blood groups (hPL mBG), and FBS, on stromal cell phenotype, genotype and function, respectively.

The intravenous application of stromal cells also bears the risk of an instant blood-mediated inflammatory reaction (IBMIR) leading to thromboembolic side effects during cell therapy [19]. Tissue factor cell surface expression seems to be a key factor, activating coagulation factor VII and finally inducing thrombin formation [20,21]. Previously, by rotational thromboelastometry (ROTEM), we found that BMSCs were less procoagulant in human plasma than stromal cells derived from white adipose tissue or umbilical cord [22]. After intravenous application, tissue factor high-expressing cord-derived stromal cells showed excessive pulmonary thromboembolism in a rat model. Such embolism risk was not observed after infusion of BMSCs with low tissue factor expression, confirming our in vitro data [22].

The aim of this study was to test the hypothesis that batch effects due to cell culture raw material precipitate culture-related trajectories in BMSC gene expression, phenotype or function. Here we tested the influence of hPL vs. FBS on BMSC transcriptomics, cell surface proteomics and function. Organoid-like cartilage disk formation was tested as a surrogate for osteo-chondral regenerative competence. The procoagulant activity of short vs. long-term cultured BMSCs, under the aegis of FBS vs. two types of hPL, respectively, was analyzed in addition by ROTEM as a marker of cell therapy hemocompatibility.

## 2. Materials and Methods

### 2.1. Bone Marrow Samples and Cell Culture

For human bone marrow mononuclear cell isolation, approval was obtained from the Institutional Review Board of the Medical University of Graz (protocol 19–252, 21–060). Samples were collected in accordance with the Declaration of Helsinki after written informed consent from healthy volunteers. Cells were cultured in alpha-modified minimum essentials Eagle’s medium, α-MEM (Sigma-Aldrich, St. Louis, MO, USA) supplemented with 5 mM N(2)-L-alanyl-L-glutamin (Dipeptiven, Fresenius Kabi, Austria), 100 U/mL penicillin and 0.1 mg/mL streptomycin (both Sigma-Aldrich). We supplemented this BMSC expansion medium additionally with 10% of either pooled hPL O/AB [23] or pooled hPL mixed blood group (mBG) in the presence of 2 IU/mL preservative-free heparin (Biochrom, Germany) [18] as indicated. An optimized concentration of 16.5% heat inactivated FBS (FBS Superior, Sigma-Aldrich) was used.

**Figure 1:**
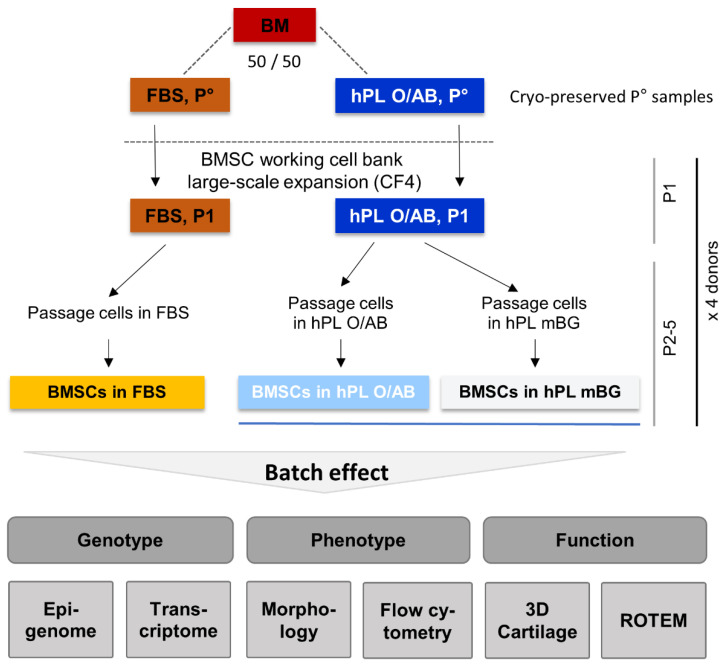
Study outline showing bone marrow stromal cell (BMSC) propagation from human bone marrow samples. Bone marrow (BM) from four healthy donors was divided 50/50 (e.g., 2 × 2.5 mL) and resuspended directly in 500 mL culture medium supplemented with either fetal bovine serum (FBS) or pooled human platelet lysate (hPL) to initiate primary culture (P°) [23]. P° and passage 1 (P1) cultures were performed with a selected lot of FBS or with hPL from blood group O donor platelets, lysed in blood group AB plasma, lacking isoagglutinins anti-A and anti-B (hPL O/AB), in four-layered cell factories (CF4), to establish a working cell bank. In subsequent cultures, hPL derived from expired platelet concentrates irrespective of the blood group (mixed blood group, mBG) was used for comparison as indicated. The batch effect of these different culture conditions on BMSC genotype, phenotype and function was studied as indicated. ROTEM, rotational thromboelastometry.

Bone marrow aspirations were divided 50/50 and stromal cells were isolated and expanded either under animal serum-free conditions with BMSC expansion media containing hPL O/AB or with media containing FBS as previously described [24] (Figure 1). We cryo-preserved BMSC passage 0 (P°, at the end of primary culture) for later analysis. For large-scale expansion, we seeded the cells directly into in four-layered cell factories (CF-4) in BMSC expansion medium supplemented with either hPL O/AB or FBS upon thawing. Expanded BMSCs from passage 1 (P1) were either analyzed or further cultured in these media until passage 4–5 (P4–5). An aliquot of BMSCs cultured in P° in hPL O/AB was transferred to BMSC expansion medium containing hPL mBG and also cultured until P4. BMSCs from P1 and P4 were further analyzed as indicated. In selected experiments, due to different cell growth of the different samples we used samples P4 +/− one passage. BMSC population doublings (PD) for all passages were calculated using the formula *LN* (fold increase cell number)/LN(2). Theoretical cumulative cell numbers were calculated, by assuming that all cells were further expanded, by multiplying the totally harvested cells of P1 with the fold-increase of the following passages P2–3 in smaller culture vessels. To quantify cell morphology, cells from donors one and two were seeded at a density of 10,000 cells/cm^2^ overnight, stained with Calcein (LIVE/DEAD™ Viability/Cytotoxicity Kit Thermo Fisher Scientific, Waltham, MA, USA) for 20 min. Images were taken at 10x magnification as four times four fields of view with an Eclipse Ti inverted microscope (Nikon, Tokyo, Japan) equipped with an automated stage (Märzhäuser Wetzlar, Wetzlar, Germany) in phase contrast and GFP channel. Individual GFP + cells were detected after thresholding using NIS AR software. Object data containing area and length were extracted for 1208–1428 individual cells and used for statistical analysis.

### 2.2. RNA-Seq, MethylCap-Seq and Bioinformatics

For both RNA sequencing (RNA-seq) and Methyl-CpG binding domain-based capture and sequencing (MethylCap-seq), libraries were sequenced on a Hiseq 4000 (Illumina, San Diego, CA, USA) with 50 bp single-end reads. We conducted quality control using FASTQC and removed residual adapter sequences and low-quality reads using Trimmomatic. We mapped reads to the Ensembl GRCh38 human genome using Bowtie 2 as described previously [9]. We submitted raw reads to the GEO database (NUMBER: GSE194303).

To analyze RNA-seq data, we calculated the number of mapped reads/gene counts using HTseq. We annotated genes using the Ensembl version 97. Expression values of protein coding genes were normalized using Deseq2 package. Principal component (PC) analysis and hierarchical clustering analysis using Euclidean distance were conducted on the whole normalized dataset to determine how samples cluster together. Differential expression analysis between different groups was conducted using Deseq2. Genes with an adjusted *p*-value < 0.05 (Benjamini-Hochberg multiple testing correction) were considered significantly differentially transcribed. Gene ontology (GO) enrichment analysis was conducted using “clusterProfiler” package in R. Benjamini–Hochberg correction was used to adjust raw *p*-values for multiple testing (adjusted *p*-value < 0.05 was considered significant). For the MethylCap-seq data, we used the QSEA R package to identify the differentially methylated regions and to build the PC analysis.

### 2.3. Phenotypic Analysis

Identity, purity, viability and morphology of BMSCs cultured under hPL (O/AB and mBG) or FBS conditions was characterized by flow cytometry using a Gallios™ (Beckman Coulter, Brea, CA, USA) as previously described [25,26].

**Figure A1:**
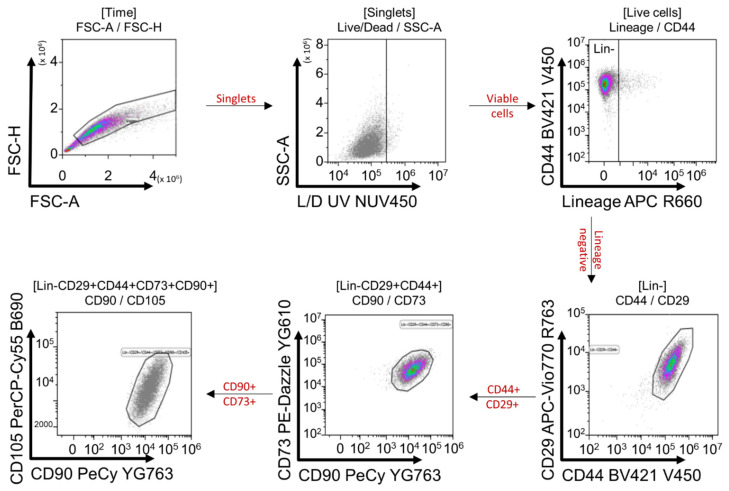
Gating strategy for cell surface marker profiling using LEGENDSCREEN™ Human PE Kit. Cells were prestained with a backbone panel (anti-human Lineage [Lin], anti-human CD29, CD44, CD73, CD90, CD105) and then analyzed with the LEGENDSCREEN™ human PE kit as follows: exclusion of nonsingle events (forward scatter area [FSC-A] versus forward scatter height [FSC-H]); gating of live cells (L/D versus sideward scatter area [SSC-A]); gating of all CD44+ and exclusion of all linage negative (versus anti-CD44); gating of all Lin-CD44+ and CD29+ (anti-CD29 versus anti-CD44); gating of all Lin-CD44+CD29+, CD90+ and CD73+ cells (anti-CD90 versus anti-CD73+); gating of all Lin-CD44+CD29+CD90+CD73+ and CD105+ (anti-CD90 versus anti-CD105)–the gated CD44+CD29+CD90+CD73+CD105+ BMSCs were used to further analyze individual target cell surface marker (anti-CD73 versus anti-target marker).

Extended flow cytometric cell surface marker screening was done using a LEGEND Screen Human phycoerythrin (PE) Kit (BioLegend, San Diego, CA, USA) according to the manufacturer’s instructions containing 347 (old kit version) or 361 (new kit version) target antibodies, respectively. All BMSCs were stained with a backbone panel containing CD14, CD19, CD31, CD34, and CD45 as lineage (Lin)-negative markers, and CD29, CD44, CD73, CD90, CD105 as positive markers, beforehand. To analyze the expression of targets, single, viable LIN^−^/CD29^+^/CD44^+^ and CD73^+^/CD90^+^/CD105^+^ cells were gated as depicted in Figure A1. Respective isotype controls were used to determine PE-labeled target antibody reactivity compared to 10 different isotypes’ control reactivity. Data were analyzed using R.

### 2.4. BMSC Function: 3D Organoid-like Cartilage Disc Formation

Chondrogenic differentiation was performed in 3D format as previously published with minor modification [7,9]. Briefly, 0.5 × 10^6^ BMSCs were seeded at indicated time points after culture onto rat collagen I-coated (C3867, Sigma Aldrich, Burlington, MA, USA) transwell inserts (3470-clear, Corning, New York, NY, USA). Cartilage disks were grown in chondrogenic differentiation medium in high glucose (4.5 g/L) DMEM (D5796, Sigma Aldrich, Burlington, MA, USA), supplemented with 40 µg/mL L-proline (P0380, Sigma Aldrich, Burlington, MA, USA), 10^−7^ M dexamethasone (#05407, Stem Cell Technologies, Vancouver, Canada), 25 µg/mL L-ascorbic acid phosphate (A8960, Sigma Aldrich, Burlington, MA, USA), insulin-transferrin-sodium selenite plus linoleic acid cell culture supplement (ITS+1, 10 ng/mL, 5.5 ng/mL, 5 ng/mL, 4.7 µg/mL, respectively; I2521, Sigma Aldrich, Burlington, MA, USA), sodium pyruvate (S8636, Sigma Aldrich, Burlington, MA, USA), L-glutamine (25033024, Thermo Fisher Scientific, Waltham, MA, USA), penicillin/streptomycin (P0781, Sigma Aldrich, Burlington, MA, USA) and 10 ng/mL transforming growth factor (TGF)-β3 (#100-36E, Peprotech, Rocky Hill, NJ, USA). We maintained cultures for 4 weeks at 37 °C by replacing 33% of the medium three times weekly. Disks were harvested, weighed after removing remaining culture medium thoroughly and subsequently formalin-fixed, paraffin-embedded and processed into 4 μm sections. Immunohistochemistry and Bern scoring [27] were done exactly as described previously [9].

### 2.5. BMSC Function: Coagulation Analysis of Early and Late BMSCs by Rotational Thromboelastometry

The procoagulant activity of BMSCs from 4 donors in plasma was analyzed by rotational thromboelastometry (ROTEM^®^, Tem^®^ International, Munich, Germany) as described previously [22]. In brief, 5 × 10^5^ BMSCs cultured in FBS and hPL O/AB (P1 and P4), and in hPL mBG (P4), respectively, were resuspended in 300 µL of citrated blood group AB plasma pooled from 10 healthy blood donors after receipt of written informed consent. After addition of 20 µL calcium chloride solution reagent (Tem^®^ International GmbH, Munich, Germany) the coagulation process was monitored for 60 min. AB plasma without cells was used as reference. The clotting time, clot formation time, maximum clot firmness and α-angle were measured in triplicate following manufacturer’s instructions. Results were analyzed according to published standards as described previously [22].

### 2.6. Statistical Analysis

Statistical analysis was done using GraphPad Prism (GraphPad Software, San Diego, CA, USA). Data are shown as mean ± standard deviation (SD). Data were analyzed for normal distribution using Shapiro–Wilk or D’Agostino and Pearson tests. Paired *t*-test, repeated measures, one-way ANOVA with multiple comparisons (Tukey) were used for disk weights, clotting time and PDs. Friedman tests with multiple comparisons (Dunn) were conducted for clot formation time. To test the impact of hPL and FBS on cumulative cell numbers and cumulative PDs, linear regression analysis was conducted using R. Significant differences were depicted as indicated in the respective figures. A *p*-value < 0.05 was considered to be significant. Statistical analysis conducted for bioinformatics was carried out using R software and as described in detail in Section 2.3 and Section 2.4, respectively.

## 3. Results

### 3.1. BMSC Expansion

We selected four healthy donor-derived BM aspirates as starting material for this study. Heparinized BM aspiration aliquots were divided in two equal parts and equilibrated directly in 500 mL BMSC expansion medium supplemented with either FBS or hPL O/AB, to initiate P°, in four-layered cell factories (CF-4) on 2528 cm^2^ growth area as described in [24,28]. Subsequent expansion was done either in FBS or in two types of hPL (O/AB vs. mBG) (Figure 1).

**Figure A2:**
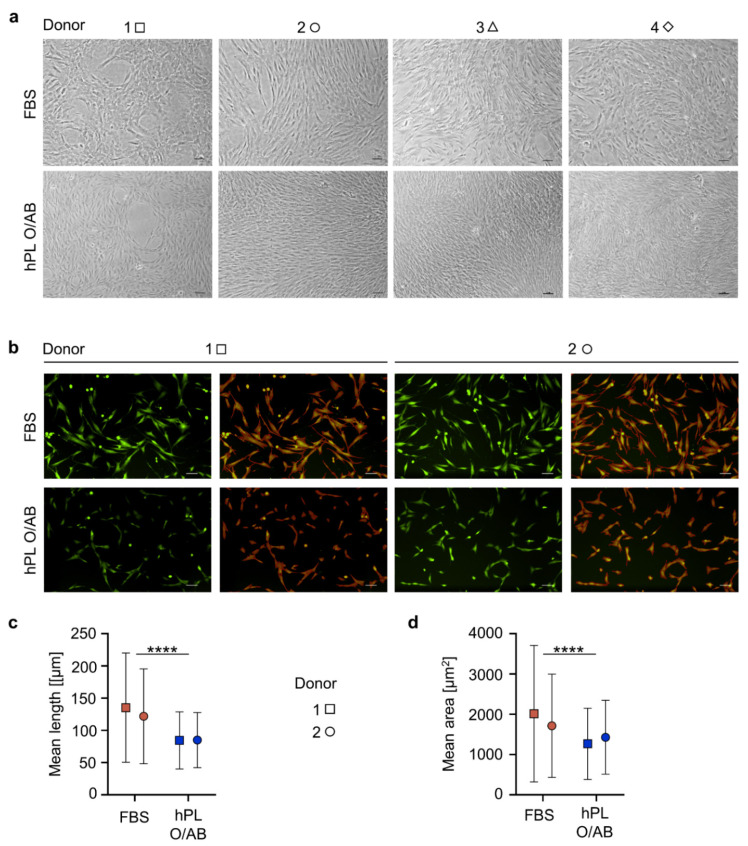
Large-scale propagation of BMSCs. (**a**) Representative cell morphology of BMSCs from four donors grown in 4-layered cell factories (CF4) with either FBS or hPL O/AB. Bright field, scale bar 100 µm (**b**) Representative pictures of calcein-stained BMSCs (green) and corresponding cell segmentation (red pseudocolor) from two donors as indicated, cultured with either FBS or hPL O/AB for 24 h at passage 1. Scale bar 100 µm (**c**) Mean length and (**d**) area of BMSCs cultured as shown in (**b**); cells analyzed for donor 1—FBS, *n* = 1428, hPL O/AB *n* = 1208; donor 2—FBS *n* = 1343, hPL O/AB *n* = 1253). Kruskal–Wallis nonparametric test (**** *p* < 0.0001).

**Figure A3:**
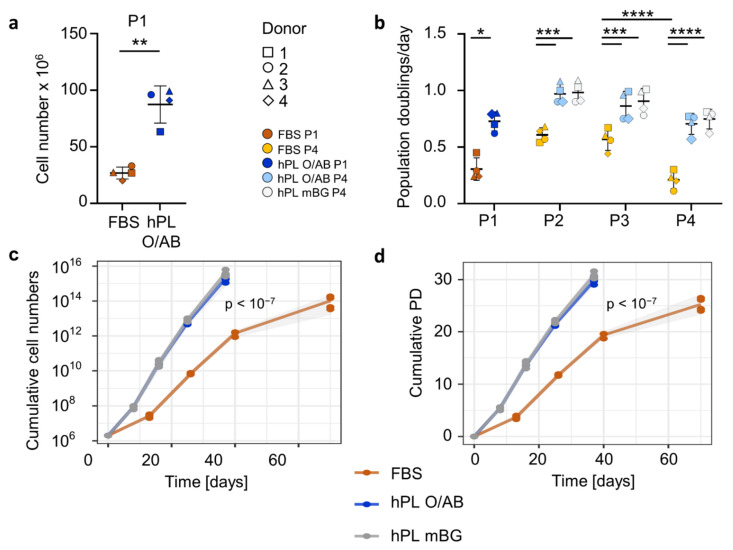
Large-scale extended propagation of BMSCs (**a**) Absolute number of BMSCs (donor 1–4, as indicated) cultured for one passage (P1) either with FBS or hPL O/AB. Paired *t*-test, ** *p* < 0.01. (**b**) Calculated population doublings per day of P1–P4 of BMSCs propagated in media containing FBS, hPL O/AB or hPL mBG. Repeated measures, one-way ANOVA with multiple comparisons (Tukey) *p* < 0.0001, *p*-values of multiple comparisons and *t*-test as depicted * *p* < 0.5, *** *p* < 0.001, **** *p* < 0.0001. (**c**) Cumulative cell numbers and (**d**) cumulative population doublings of BMSCs cultured in different media over time. Grey ribbon indicates 95% confidence intervals. Linear regression analysis showed significant differences between FBS and hPL medium with *p* < 10^−7^ for both cumulative cell counts and cumulative PD. No significant differences were found between hPL O/AB and hPL mBG; *n* = 4. Abbreviations: P—passage; FBS—fetal bovine serum; hPL—human platelet lysate; O/AB—blood group O platelets lysed in AB plasma; mBG—mixed blood group of platelets lysed in their original plasma.

We confirmed previous observations that hPL-expanded BMSCs displayed slimmer cell shape [24], reached a higher cell density (Figure A2) and a significantly enhanced proliferation resulting in a mean 3.3-fold higher cell number already after first passage (P1; Figure A3b) [25]. Microscopy measurements confirmed a significantly lower mean cell length and significantly lower mean cell area of hPL-expanded cells (Figure A2). Beginning at P2, BMSCs were seeded at each passage exactly at 100 cells/cm^2^ in cell culture flasks to create standardized high proliferation exhaustive culture conditions [29,30]. The low seeding density culture strategy resulted in a significantly reduced population doubling time of as short as 24 h in hPL-supplemented cultures compared to mean 40 h in FBS cultures. Maximum population doublings were observed at P2 and P3 for all culture batches in hPL and FBS, respectively. A significant decline in proliferation, reminiscent of proliferative senescence, was evident at P4 for FBS cultures (Figure A3a), confirming previous observations [30]. Calculated cumulative mean cell numbers were 1.98 ± 0.81 × 10^15^ and 4.06 ± 1.60 × 10^15^ for the two independent hPL-driven cultures, corresponding to 29.8 ± 0.6 as well as 30.8 ± 0.5 population doublings within only 37 ± 3 days in hPL mBG and hPL O/AB, respectively. FBS cultures yielded 29.6-fold less cells with a calculated maximum of 0.10 ± 0.07 × 10^15^ cells, corresponding to 25.2 ± 1.2 population doublings within 70 ± 9 days (all numbers mean ± SD; Figure A3c,d). We summarized the experimental strategy and the color code used throughout the study in Figure 1.

### 3.2. Transcriptome and Methylome Characterization

**Figure 2:**
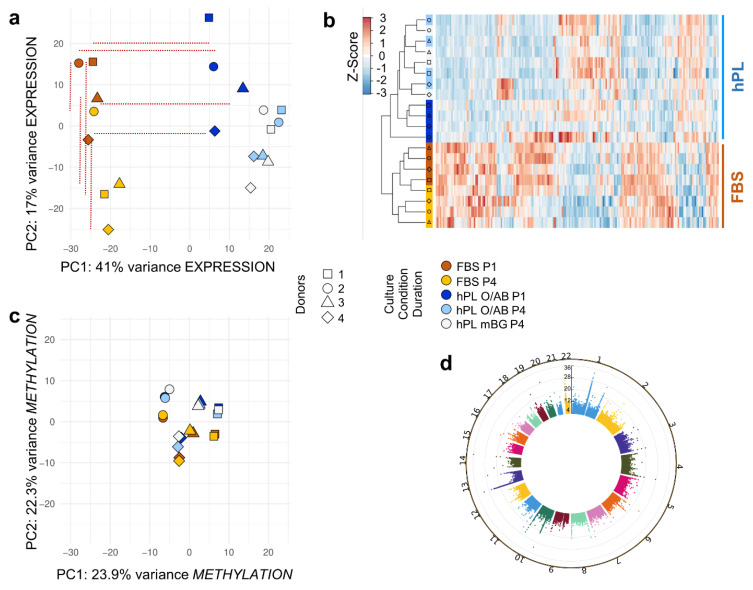
Batch effects on culture-expanded BMSC transcriptomics ((**a**) + (**b**)) and methylomics ((**c**) + (**d**)): (**a**) Principal component analysis (PCA) showing the spatial clustering of the different samples (FBS, ocher shades; hPL, blue shades; P1, darker color; P4, lighter colors; hPL mBG open symbols) and different donors (symbols as indicated; color code and symbol legend for **a**–**c**). The culture conditions (hPL-vs. FBS-supplemented) explained most of the variance in the dataset (PC1, 41%). Horizontal red dotted lines highlight the distance of samples from the same donor cultured with FBS (left) vs. hPL (right). PC2 separated samples due to culture duration and donor variation. Vertical dotted lines highlight distance between P1 and P4, regardless of donor variation. (**b**) Heatmap with expression values of top 100 most variable genes (see Figure A4). Samples in rows as indicated and genes in columns; different batch-defining serum supplements and passages in the general color code. Gene expression values were column Z-score normalized; lower expression blue and higher expression red color as shown in the legend. (**c**) PCA of differentially methylated regions with PC1 covering 23.9% and PC2 22.3% of differences. Color and symbol code as indicated. (**d**) Differentially methylated regions were depicted as dots on the Manhattan plot for all autosomal chromosomes. The dot height corresponds to the level of significance with higher dots indicating higher significance, i.e., lower *p*-value (-Log10 adjusted *p*-values).

Gene expression profiling in BMSC samples from four healthy donors was performed by RNA-seq at early (P1) and late time points (P4) after culture in either hPL- or FBS-supplemented media, respectively. Culture conditions, i.e., hPL vs. FBS, were the major driver of variation. An impressive batch effect of culture conditions was observed already after P1 separating hPL-vs. FBS-driven cultures into trajectories due to 41% of the gene expression variance in principle component 1 (PC1). This separating batch effect of culture conditions persisted over time of culture. Differences in PC2 explaining 17% of the total variance mainly corresponded to donor variation and culture duration (Figure 2a).

**Figure A5:**
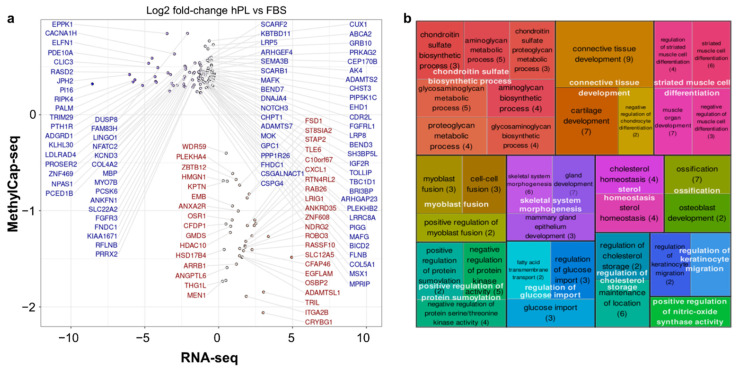
Overlap between RNA-seq and MethylCap-seq. (**a**) Genes found significantly upregulated and hypomethylated in hPL vs. FBS (red labels) or downregulated and hypermethylated in hPL vs. FBS (blue labels). X axis shows the log2-fold changes for RNA-seq. Y axis shows log 2-fold methylation changes. (**b**) Gene ontology (GO) enrichment analysis showing significant biological processes enriched for genes found downregulated and hypermethylated in hPL vs. FBS (blue labelled genes, in a) (adjusted *p*-value < 0.05, Benjamini–Hochberg multiple testing correction). Similar GO categories are clustered together and labelled based on semantic similarity. The size of boxes is related to the number of genes in the respective category.

Hierarchical clustering of the 100 most variable genes in a heatmap showed that samples within hPL vs. FBS batches cluster together regardless of culture duration (Figure 2b and Figure A2). Whole genome methylation analysis by MethylCap-seq revealed that both donor variation and hPL vs. FBS serum component differences during propagation contribute to variation in the DNA methylation pattern. Concerning the two most prominent components, represented by PC1 and PC2, the four donor-derived methylation datasets clustered together in PC1 more than PC2. The hPL- vs. FBS-driven culture conditions separated methylation patterns for all four donors, mainly in PC2. Minor differences were detectable comparing hPL O/AB with mixed blood group hPL (Figure 2c). We found most significant DNA methylation changes on chromosomes 1, 8, 10 and 12 (Figure 2d). We observed an overall gain of methylation in FBS compared to hPL with more than 21% of CpG islands being significantly hypermethylated in FBS while 12.5% were hypomethylated. By investigating the overlap between RNA-seq and MethylCap-seq, we identified 100 genes significantly downregulated and hypermethylated, and 38 genes significantly upregulated and hypomethylated, in hPL vs. FBS. GO term enrichment analysis conducted for the 100 genes downregulated and hypermethylated in hPL showed enrichment of GO categories including connective tissue development and striated muscle cell differentiation. This included reduced expression of genes negatively affecting chondrogenesis or genes involved in more pleiotropic functions such as *ADAMTS7* and *RFLNB* (GO “negative regulation of chondrocyte differentiation”). No GO terms were found significantly enriched in the 38 genes upregulated and hypomethylated in hPL vs. FBS. (Figure A5).

**Figure 3:**
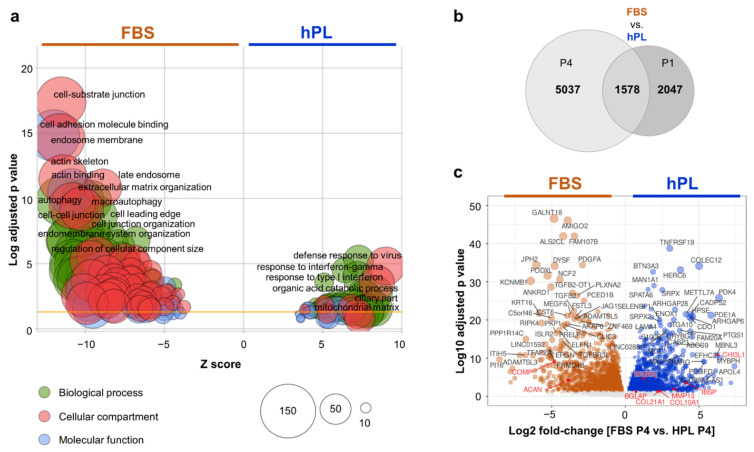
Predominantly regulated pathways. (**a**) Gene Ontology (GO) enrichment analysis comparing hPL-(right side) or FBS-driven cultures (left side). We labelled only the top 12 FBS- and, with significantly lower adjusted *p*-value, top 6 hPL-enriched GO terms. A higher absolute Z-score indicates a stronger level of enrichment. The yellow line indicates the adjusted *p*-value of 0.05. Biological process, cellular component and molecular function GO categories are highlighted in green, red and blue, respectively. Circle size represents number of genes found in the respective GO term category. Only genes commonly differentially regulated and showing the same direction (both passages UP or DOWN in hPL) irrespective of culture duration (1540 out of 1578 in the central component, in (**b**), were analyzed. (**b**) Venn diagram showing the number of differentially expressed genes in cells obtained by hPL- vs. FBS-driven culture for P4 and hPL vs. FBS for P1 (adjusted *p*-value < 0.05). (**c**) Volcano plot showing most significantly expressed genes in FBS- (left) and hPL-driven cultures (right). Red dots marking genes significantly differentially expressed (adjusted *p*-value < 0.05); black dots, not significant. Most significantly differentially expressed genes indicated by gene ID.

The GO enrichment results of commonly differentially expressed genes during BMSC culture draw a clearly accentuated picture (Figure 3a). Comparing gene expression of BMSCs expanded in FBS vs. hPL displayed 3,625 significantly differentially regulated genes after P1 and 6615 differentially expressed genes after P4 suggesting progressive differences in response to extended cell culture. Interestingly, 1578 genes were detected commonly differentially transcribed in FBS vs. hPL-driven cultures irrespective of culture duration (Figure 3b). Among these 1578 genes, 1540 showed similar profiles (UP or DOWN in both passages), including 993 genes significantly upregulated in FBS compared to 547 in hPL. This difference is also reflected in the enrichment analysis where a higher number of 124 GO categories were enriched in FBS compared to 54 in hPL. We found strong enrichment of genes categorized as interferon-response-related (*n* = 20) and antiviral-response-related (*n* = 54) as well as energy metabolism responses (*n* = 33) in hPL-driven cultures (Supplementary Table S1).

Besides being upregulated in hPL, the immune-response-related genes, *CD40*, *CD74, APOL4* and *HLA-DRB1,* also showed a strong positive correlation with cartilage disk weights (see also Figure A7b). The FBS-expanded BMSCs generally deviated in their cytoskeleton and extracellular matrix responses as well as adhesion and cell junction gene expression (Figure 3a). Key chondrogenic markers were found significantly upregulated in hPL such as *RUNX2* (Log2 fold-change, FC 0.76, P1 and 1.27, P4), *MMP13* (Log2 FC 3.65, P1 and 3.27, P4), *BGLAP* (Log2 FC 1.83, P1), *CHI3l1* (Log2 FC 2.91, P1 and 6.23, P4), *COL10A1* (Log2 FC 2.37, P4), *IBSP* (Log2 FC 4,12, P4), *COL21A1* (Log2 FC 2.19, P4), respectively. *RUNX2* also showed a strong positive correlation between RNA-seq expression and the cartilage disk weights (R^2^ = 0.47, *p* < 0.05). Genes downregulated in hPL such as *COMP* (Log2 FC −4.28, p1 and −4.83 P4) and *ACAN* (Log2 FC –4.36, P1 and –3.87, P4) (Figure 3c).

### 3.3. Polychromatic Flow Cytometry

**Figure 4:**
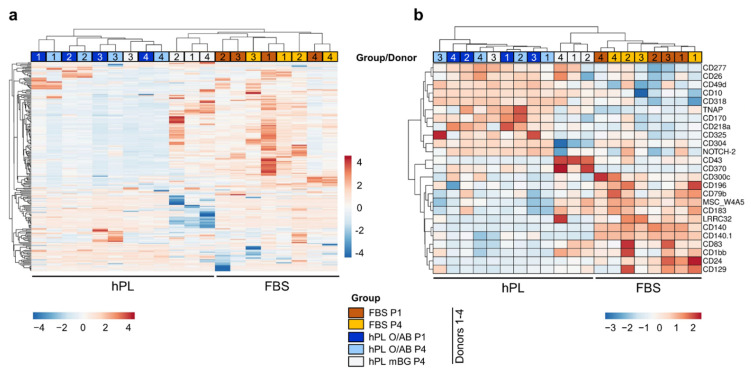
Cell surface marker profiling of BMSCs. (**a**) Hierarchical clustering heatmap showing relative cell surface marker expression of all samples and culture conditions as indicated, based on isotype control staining of the corresponding target antibody. Individual marker expressions were row Z-score normalized. Lower expression in blue and higher expression in red as indicated by color. Gating strategy depicted in Figure A1. (**b**) Hierarchical clustering heatmap of cell surface markers that exhibited significant median differences of at least 10% between BMSCs in hPL O/AB and FBS.

**Figure A6:**
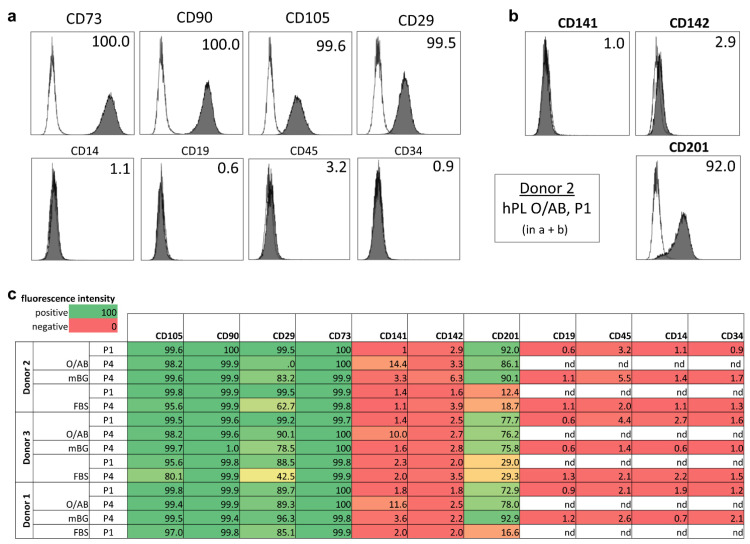
BMSC phenotype, identity and purity. (**a**) Representative (donor 2 P1, hPL O/AB) flow cytometry histograms showing target antibody reactivity as indicated (in grey), compared to isotype control reactivity (open histograms) measured on 10,000 viable cells. (**b**) Coagulation factor III, thrombomodulin and endothelial protein C receptor (EPCR) reactivity of donor 2, P1 in hPL O/AB. (**c**) Multicolor flow cytometry analysis summary of three donors at P1 and P4 after culture as indicated in hPL (O/AB vs. mBG) or FBS.

After confirming identity (<5% hematopoietic contamination CD14/19/34/45) and purity (>95% CD73/90/105/29) of the expanded BMSCs [31] after P1, using conventional multicolor flow cytometry (Figure A6), we performed a >300 immune-related cell marker LEGEND screen with stringent gating strategy (Figure A1). Heatmap analysis, providing an overview of multiplexed data, allowed the display of clear marker expression trajectories. In this display, BMSCs expanded in hPL O/AB clustered together as did those expanded in FBS. Marker profiles in hPL O/AB cultures at early (P1) and late time points (P4) were more adjacent indicating higher heterogeneity among FBS-expanded BMSCs. Surprisingly, three of four hPL mBG-expanded donors clustered together with their FBS-expanded counterparts at P4. Large cohorts of clearly differentially expressed markers were identified (Figure 4a). In a detailed analysis, we identified numerous immunity-related marker molecules to be differentially expressed by hPL O/AB- vs. FBS-expanded BMSCs further supporting the hypothesis that batch effects precipitate culture-related gene expression trajectories (Figure 4b).

### 3.4. Organoid-like 3D Cartilage Disk Formation

**Figure 5:**
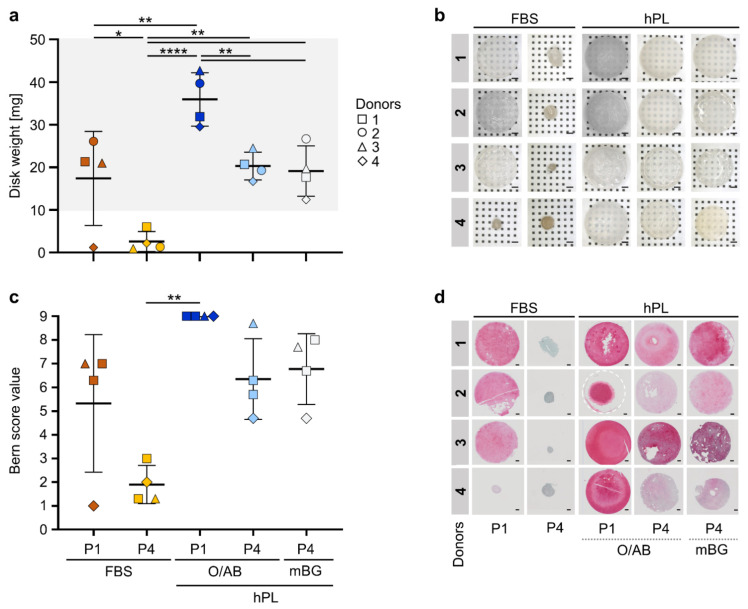
Organoid-like 3D cartilage disk formation. (**a**) Weight of 3D cartilage disks generated in triplicates from BMSCs of four donors (1–4, depicted with symbols as indicated), pre-expanded in different media, and differentiated after P1 or P4 as indicated. One to three representative disks per sample were fixated and weighed and the remaining two discs were snap frozen for further analysis. Proper disk formation resulted in disks > 10 mg (grey area). Repeated measures, one-way ANOVA with multiple comparisons (Tukey), *p*-values of multiple comparisons as depicted (* *p* < 0.5, ** *p* < 0.01, **** *p* < 0.0001). (**b**) Representative pictures of corresponding fixated 3D cartilage disks created from BMSCs of donors 1–4 after hPL- or FBS-expanded early (P1) and late passage (P4) cultures as indicated. Scale bar 1 mm; *n* = 4. (**c**) Bern scoring of stained cartilage disk sections by three individual experts in blinded fashion; mean ± SD results, statistics as in (**a**), *n* = 4. (**d**) Representative corresponding Safranin O/Fast Green staining results depicted as indicated. Entire disk sections shown except for donor 2 hPL O/AB where curved structure disabled perfect sectioning and disk margin was illustrated by a white hatched line. Scale bar 1 mm.

**Figure A7:**
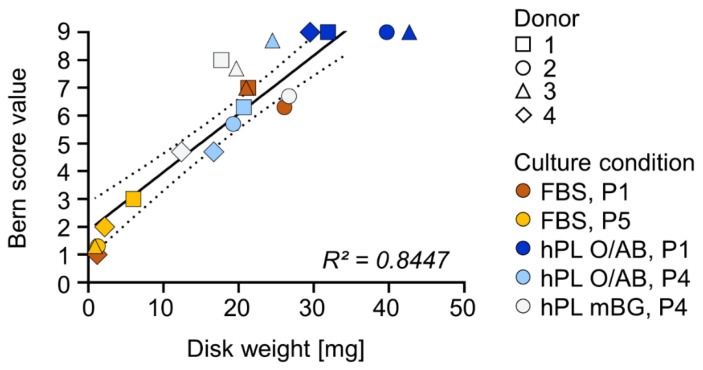
Correlation of cartilage weight with Bern score and proteoglycan content. Weight of 3D cartilage disks generated in triplicate from BMSCs of four donors (donor 1–4, depicted with symbols as indicated), cultured in different conditions and differentiated after different passages (see legend for color code) correlated to Bern score values of the same samples. Linear regression analysis, *p* < 0.0001, *n* = 20.

To validate skeletal regenerative function of the different early and late cell products we selected organoid-like 3D cartilage disk formation as one of the most stringent readouts for endochondral differentiation [7,9]. All hPL- and three of four FBS-driven P1 cell products displayed competence to form 3D cartilage discs under appropriate culture conditions. BMSCs derived from hPL-driven cultures were significantly more potent than cells from FBS cultures, producing more than twice as heavy cartilage disks. All P4 hPL-expanded cell products, made of cells beyond 29 PDs, showed significantly reduced weight of the end-products after 3D chondrogenesis. The hPL O/AB- and hPL mBG-expanded late cell products generated mean 20.3 ± 3.3 and 19.1 ± 5.9 mg weight disks, respectively. The FBS-expanded P4 cell products lost their capacity to form cartilage disks but just gathered into 2.6 ± 2.3 mg (mean ± SD) condensed aggregates (Figure 5a,b). Bern scoring of in vitro engineered cartilage [27] showed significant loss of cartilage quality in P4 FBS-expanded cultures (Figure 5c) with a highly significant correlation between Bern score and cartilage disk weight (R^2^ = 0.8447; Figure A7). Safranin O plus Fast Green staining of sectioned disks or cell aggregates confirmed these findings (Figure 5d).

### 3.5. Procoagulant Activity of BMSCs

**Figure 6:**
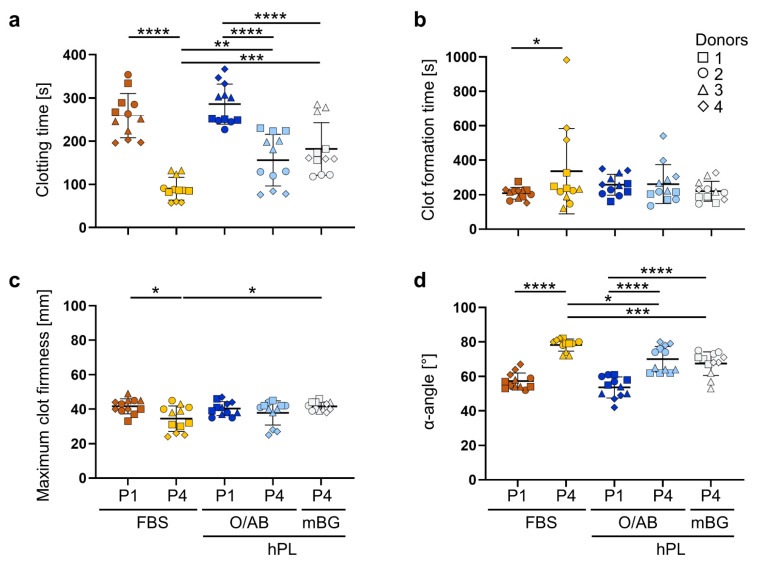
Coagulation activity of early (P1) and late passage (P4) BMSCs. (**a**) Significantly shortened clotting time values after 4 passages in FBS, more than hPL O/AB and hPL mBG, compared to passage 1, respectively. Repeated measures, one-way ANOVA with multiple comparisons (Tukey). (**b**) The clot formation time and (**c**) maximum clot firmness showing limited donor dependent differences. Clot formation time: Friedman test with multiple comparisons (Dunn); maximum clot firmness ANOVA as in (**a**). (**d**) Significantly increased α-angles resulting from higher procoagulant activity of all late (P4) BMSCs, compared to early (P1) BMSCs; statistics as in (**b**). Results of 4 donors analyzed in triplicate, symbols as indicated; p-values of multiple comparisons as depicted—* *p* < 0.5, ** *p* < 0.01, *** *p* < 0.001, **** *p* < 0.0001, ns, not significant.

Thromboembolism is a feared risk of cell-based therapies, particularly triggered by extrahematopoietic cells after extended culture [22]. In a second set of functional experiments, we therefore measured the procoagulant capacity of early and late BMSC culture products expanded in FBS, hPL O/AB or hPL mBG, by ROTEM. BMSCs were resuspended in citrated AB plasma. After addition of CaCl_2_, the clotting reaction was monitored for 60 min. The time until clot formation initiation (amplitude of 2 mm, ‘clotting time’), the time from initiation of clot formation until a 20 mm amplitude of clotting (‘clot formation time’), the peak of the amplitude (‘maximum clot firmness’) and the kinetics of the clotting represented as ‘α-angle’ were compared. The clotting time and the α-angle of P1 BMSCs did not differ significantly, irrespective of culture in FBS or hPL O/AB. Extended culture (P4) resulted in significantly shortened clotting time with changed clotting kinetics (Figure 6a,d).

After extended in vitro culture, measured at the end of P4, BMSCs showed a significantly shortened clotting time and increased α-angle reflecting a higher procoagulant activity acquired in all media conditions compared to P1. Notably, the clotting risk was most pronounced after FBS-compared to hPL-driven propagation. Minor variations in the clot-formation time and maximum clot firmness were donor-dependent rather than serum-dependent (Figure 6b,c).

## 4. Discussion

Batch effects are evident in most complex analytic assay formats but are mostly neglected in cell manufacture. Here, we demonstrate profound batch effects on BMSC gene expression, phenotype and function, detected already after just one passage of large-scale preclinical cell propagation. We confirmed our historical results showing significantly superior BMSC multiplication in the presence of hPL compared to FBS [24,28,30,32,33].

Transcriptomics comparing FBS- vs. hPL-expanded BMSCs displayed distinct gene expression trajectories due to 3625 significantly differentially expressed genes in P1. Progressive differences in 6,615 differentially expressed genes were observed after four culture passages at low seeding density, i.e., augmented proliferation of up to 30 PDs, representing approximately 1 billion-fold expansion in hPL. The culture conditions played a greater role for the variance compared to donor variation. Interestingly, BMSCs in FBS showed an enrichment for certain GOs related to cartilage development. Chondrogenic markers *COMP* and *ACAN* were found to be upregulated in FBS. Key chondrogenic markers such as *RUNX2* (osteochondro progenitor transcription factor), *COL10A1*, *MMP13* (both hypertrophic chondrogenesis markers), *BGLAP* and *CHI3L1* were found to be significantly upregulated in hPL. A similar observation was made previously [34] where *ACAN* expression was found to be strongly upregulated in FBS while *SOX9*, *RUNX2* and *ALP* were found to be upregulated in hPL. We found higher values of *SOX9* and *ALP* in hPL as well, but these were not significant due to high variability. Published data indicate that hPL maintained cells at an earlier stage of differentiation in terms of chondrogenesis, compared to FBS driving a more committed differentiation state [34].

BMSCs expanded in hPL showed an induction of genes related to interferon gamma (IFN-γ) and immune response gene categories in our study. While it is not yet clear how such response may influence cartilage development, a previous study indicated that IFN-γ could enhance chondrogenesis and anti-inflammatory activity [35] IFN-γ was also shown to be involved in the immune response of diseased cartilage [36,37].

We also found deviations in methylomics separating samples by donor origin and culture conditions in hPL vs. FBS, but barely by culture duration. It has also been shown that hPL-expanded cells displayed lower levels of senescence compared to cells expanded in FBS [38]. During senescence, epigenetic alterations and notably DNA methylation play an important role in shaping the expression of proliferation-related genes [39]. DNA methylation changes in long-term cultured cell lines are well established [40,41]. Methylation changes during long-term stromal cell culture were recently discovered to be mainly due to epigenetic drift, i.e., not directly regulated by a targeted mechanism [42]. Moreover, DNA methylation is a key epigenetic modification for the establishment and maintenance of cellular identity [43]. We observed an overall gain of methylation in FBS-expanded BMSCs suggesting a different aging process between the different cultures. We found limited overlap between the RNA-seq and the MethylCap-seq affecting 138 genes. This may suggest that other mechanisms, histone modification or nonepigenetic changes, may play a role. Interestingly, 100 of these overlapping genes were downregulated and hypermethylated in hPL compared to FBS and showed a clear enrichment for genes related to skeletal development. Strikingly, part of these genes belongs to GO category “negative regulation of chondrocyte differentiation” including *RFLNB* and *ADAMTS7.* A previous study showed that forced expression of *ADAMTS7* and *ADAMTS12* can suppress differentiation of uncommitted mesenchymal cells to the chondrocyte lineage [44]. Another gene, *FGFR3* can act as negative regulator of chondrogenesis [45].

The canonical fibroblastic phenotype of BMSCs persisted over the observation period. However, we discovered a significant differential expression of hundreds of marker molecules in an advanced LEGEND screen comparing hPL- vs. FBS-expanded BMSCs. Selected immune function-related differentially expressed molecules on hPL O/AB-expanded BMSCs, after just one culture passage, included the extended B7 superfamily member CD277 (butyrophilin, BNT3A1). It is implicated in mammalian and microbial phosphoagonist cellular stress sensing in human γδT cells [46]. Several additional significantly upregulated surface molecules predominantly relate to stem cell and immune cell interaction, including the T cell activator CD26 (dipeptidylpeptidase IV; ADA receptor) [47], CD49d (α4 integrin, CD106/VCAM-1 ligand) [48], CD10 (endopeptidase; sphere-forming and perivascular calcifying cell marker) [49,50], CD318 (CUB domain-containing protein, CD6 ligand) [51], CD170 (Siglec-5) [52], CD325 (N-cadherin) [53], CD304 (neuropilin-1) [54] and NOTCH-2 [55]. BMSCs from three of four donors showed distinct upregulation of CD43 [56] and the dendritic cell marker CD370 [57] after expansion in hPL mBG (P4) and clustered together with their FBS-expanded counterparts. Selected surface molecules upregulated on BMSCs by FBS culture predominantly included cytokine receptors CD129 (IL-9 receptor) [58], CD140 (PDGF receptor) [59], and the chemokine receptors CD183 (CXCR3) as well as CD196 (CCR6) [60], among others. Better understanding of these differential signatures may help to explain the differences in immunomodulatory capacity of stromal cells, which were previously mainly attributed to organ and donor variability [11].

**Figure A8:**
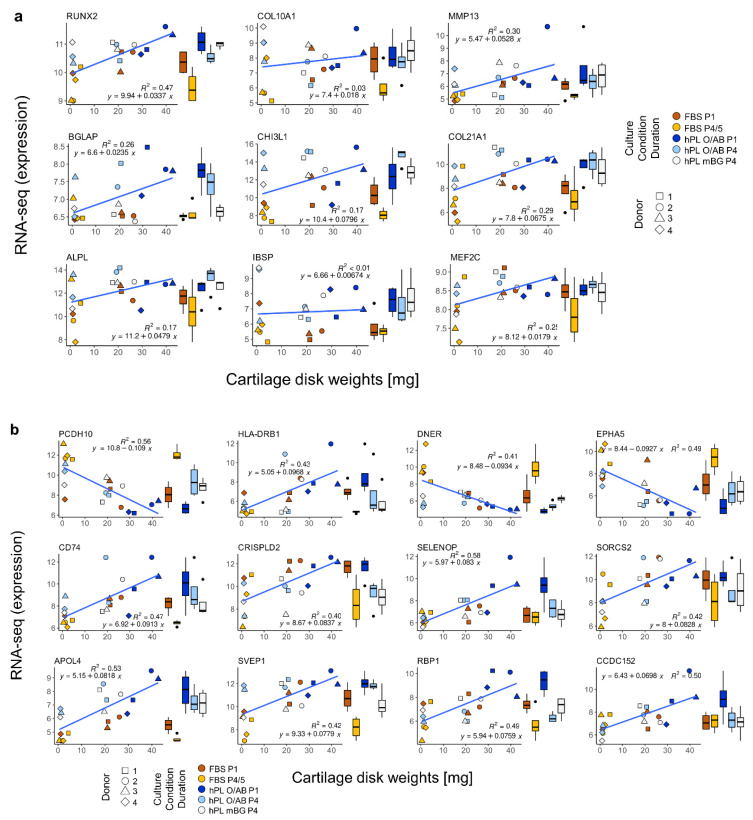
Correlation analysis between RNA-seq gene expression and cartilage disk weight. For each gene, a dual plot shows, on the left side, the correlation analysis between RNA-seq expression on the *Y*-axis and cartilage disk weights on the *X*-axis, and on the right side, box plots depicting the RNA-seq signal in the different culture condition. Different donors are depicted by different symbols and culture conditions by different colors, as indicated. A blue linear-regression line is shown together with the regression equation and R2 value. (**a**) Selected genes linked to chondrogenesis. (**b**) Top 12 genes with the highest absolute regression coefficient. Linear regression analysis of differentially transcribed genes was conducted and genes showing significant *p* < 0.05 and R^2^ > 0.40 were selected.

Cartilage regeneration represents one of the prime pursued applications for BMSCs [61]. Cell expansion is a prerequisite for most stromal cell therapies due to the limited availability of primary cells [62]. In this study, we extended previous observations related to hPL vs. FBS serum supplements [7,63] in a head-to-head comparison of paired oligoclonal BMSC preparations from the same donor. We found that, already after one culture passage, hPL-expanded BMSC were significantly more potent than FBS-expanded BMSCs from the same donor in generating significantly larger 3D cartilage disks despite equal starting cell number. Bern scoring [27] indicated a significantly improved engineered cartilage quality. More impressively, FBS-expanded BMSCs lost their chondrogenic potential after excessive expansion, i.e., >20 population doublings. The hPL-expanded BMSC maintained chondrogenesis despite reduced cartilage disk weight but still displayed intact disk shape and engineered cartilage quality. These results argue in favor of using hPL for BMSC propagation for skeletal regeneration. Interestingly, we also showed that donor variation may have an impact on cartilage formation irrespective of culture in both FBS and hPL media. We were still able to produce cartilage disks with hPL- but not FBS-expanded BMSCs from one of four donors. We may speculate how the early transcriptomic and/or phenotypic batch effects translate into the subsequent significant differences in 3D chondrogenesis. We identified a number of genes showing strong positive correlation with 3D cartilage disk weight, including immune-related (*HLA-DRB*1 and invariant chain, *CD74*) metabolism-related (selenoprotein P, *SELENOP*; sortin-related receptor, *SORCS2*), morphogens (cysteine rich secretory protein, CRISPLD2, bone morphogenic protein-inducible protein and chitinase 3-like 1 chondrocyte protein, *CHI3L1*), apolipoprotein A4 (*APOL4*), delta and notch-like epidermal growth factor-related receptor (*DNER*) as well as *RUNX2* (Figure A8a,b). *HLA-DRB1, CD74* and *APOL4* were also found previously to be induced by IFN-γ in osteoarthritis chondrocytes [64]. *DNER* was shown to be negatively correlated with chondrogenic potential [65] Additional research is required to better understand the contribution of individual differentially regulated genes and the resulting signatures on BMSC function also in relation to the batch effects observed.

We selected clotting risk analysis as a second functional readout because intravascular application of tissue factor expressing cells, i.e., virtually all extrahematopoietic cell products, can result in an instant blood-mediated inflammatory response (IBMIR) [66]. It is not clear yet if unintended blood contact after local injection can also result in clotting-related side effects [67,68]. ROTEM results revealed a culture-induced increase of the procoagulant activity of BMSCs in AB plasma, reflected by significantly shortened clotting time values after extended culture (P4). Higher procoagulant activity of all P4 BMSCs with a statistically significant increase of α-angles was observed compared to early cells, indicating negative impact of extended culture and exhaustive propagation. The limited clotting risk of short-term expanded BMSCs confirmed earlier results [22,69]. Another important aspect is the capacity of the generated cartilage to adhere to the disease site. Although we have not conducted adhesion strength assays in our study, a systematic review suggested that hPL was as effective as FBS in promoting adhesion [70].

In the course of this study, we observed an unexpectedly distinctive serum-related batch effect resulting in pronounced differences in gene expression and phenotype as well as corresponding differences in BMSC chondrogenic function. This has broader implications, which are not restricted to skeletal regeneration. The distinct immune-related phenotypic changes may help to select most potent manufacturing conditions for immunomodulatory stromal cells. Marker profiling as indicated in this study and corresponding functional assays will allow the development of predictive potency assays for cell-based therapeutics [71]. Future studies need to address whether batch effects are restricted to serum components or if additional factors, particularly complex undisclosed proprietary ingredients in defined media, also elicit batch effects. There is an additional critical need for developing strategies for nonhealing bone fractures. In this work, we provided evidence that hPL provides a better environment for BMSCs regarding cartilage formation. Based on the endochondral bone formation capacity of BMSCs [7] these cartilage disks might also represent templates for endochondral bone formation.

## 5. Conclusions

Batch effects occur during cell therapy manufacture. Signatures identified in this study will help to develop sensitive potency assays to guarantee stringent release criteria for cell therapeutics.

## Figures and Tables

**Figure A4:**
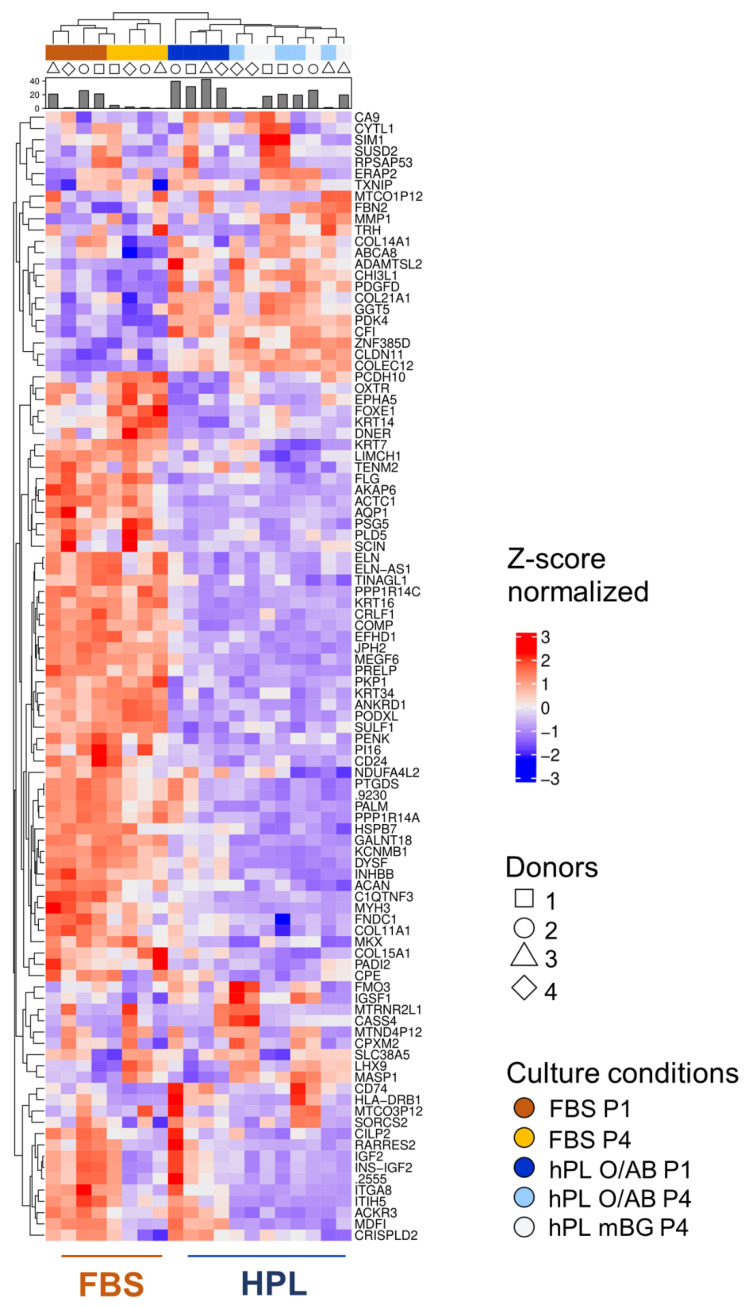
Hierarchical clustering heatmap with expression values of the top 100 most variable genes. Gene expression values were row Z-score normalized where lower expression is denoted by blue and higher expression by red color as shown in the legend above. FBS samples cluster together and HPL samples cluster together, passages have less influence.

## Data Availability

We submitted raw reads of RNA-seq and MethylCap-seq to the GEO database (NUMBER: GSE194303).

## Supplementary Materials

The following supporting information can be downloaded at: https://www.mdpi.com/article/10.3390/cells11060946/s1, Table S1: List of genes in alphabetical order related to viral response, metabolism or interferon response found significantly upregulated in hPL compared to FBS.

## References

[1] Huey D.J., Hu J.C., Athanasiou K.A. (2012). Unlike bone, cartilage regeneration remains elusive. Science.

[2] Murphy M.P., Koepke L.S., Lopez M.T., Tong X., Ambrosi T.H., Gulati G.S., Marecic O., Wang Y., Ransom R.C., Hoover M.Y. (2020). Articular cartilage regeneration by activated skeletal stem cells. Nat. Med..

[3] Matsushita Y., Nagata M., Kozloff K.M., Welch J.D., Mizuhashi K., Tokavanich N., Hallett S.A., Link D.C., Nagasawa T., Ono W. (2020). A Wnt-mediated transformation of the bone marrow stromal cell identity orchestrates skeletal regeneration. Nat. Commun..

[4] Monaco G., Ladner Y.D., El Haj A.J., Forsyth N.R., Alini M., Stoddart M.J. (2021). Mesenchymal Stromal Cell Differentiation for Generating Cartilage and Bone-Like Tissues In Vitro. Cells.

[5] Murdoch A.D., Grady L.M., Ablett M.P., Katopodi T., Meadows R.S., Hardingham T.E. (2007). Chondrogenic differentiation of human bone marrow stem cells in transwell cultures: Generation of scaffold-free cartilage. Stem Cells.

[6] Frerker N., Karlsen T.A., Lilledahl M.B., Brorson S.H., Tibballs J.E., Brinchmann J.E. (2021). Scaffold-Free Engineering of Human Cartilage Implants. Cartilage.

[7] Reinisch A., Etchart N., Thomas D., Hofmann N.A., Fruehwirth M., Sinha S., Chan C.K., Senarath-Yapa K., Seo E.Y., Wearda T. (2015). Epigenetic and in vivo comparison of diverse MSC sources reveals an endochondral signature for human hematopoietic niche formation. Blood.

[8] Reinisch A., Thomas D., Corces M.R., Zhang X., Gratzinger D., Hong W.J., Schallmoser K., Strunk D., Majeti R. (2016). A humanized bone marrow ossicle xenotransplantation model enables improved engraftment of healthy and leukemic human hematopoietic cells. Nat. Med..

[9] Hochmann S., Ou K., Poupardin R., Mittermeir M., Textor M., Ali S., Ellinghaus A., Jacobi D., Elmige R.J.A.J., Donsante S. The enhancer landscape predetermines the skeletal regeneration capacity of stromal cells.

[10] Goh W.W.B., Wang W., Wong L. (2017). Why Batch Effects Matter in Omics Data, and How to Avoid Them. Trends Biotechnol..

[11] Ketterl N., Brachtl G., Schuh C., Bieback K., Schallmoser K., Reinisch A., Strunk D. (2015). A robust potency assay highlights significant donor variation of human mesenchymal stem/progenitor cell immune modulatory capacity and extended radio-resistance. Stem Cell Res. Ther..

[12] Beachy S.H., Wizemann T., Hackmann M., National Academies of Sciences, Engineering, and Medicine (2019). Exploring Sources of Variability Related to the Clinical Translation of Regenerative Engineering Products: Proceedings of a Workshop.

[13] Zhang C., Zhou L., Wang Z., Gao W., Chen W., Zhang H., Jing B., Zhu X., Chen L., Zheng C. (2021). Eradication of specific donor-dependent variations of mesenchymal stem cells in immunomodulation to enhance therapeutic values. Cell Death Dis..

[14] Leek J.T., Scharpf R.B., Bravo H.C., Simcha D., Langmead B., Johnson W.E., Geman D., Baggerly K., Irizarry R.A. (2010). Tackling the widespread and critical impact of batch effects in high-throughput data. Nat. Rev. Genet..

[15] EMA (2007). Guideline On Human Cell-Based Medicinal Products.

[16] Strunk D., Lozano M., Marks D.C., Loh Y.S., Gstraunthaler G., Schennach H., Rohde E., Laner-Plamberger S., Oller M., Nystedt J. (2018). International Forum on GMP-grade human platelet lysate for cell propagation: Summary. Vox Sang..

[17] Schallmoser K., Henschler R., Gabriel C., Koh M.B.C., Burnouf T. (2020). Production and Quality Requirements of Human Platelet Lysate: A Position Statement from the Working Party on Cellular Therapies of the International Society of Blood Transfusion. Trends Biotechnol..

[18] Oeller M., Laner-Plamberger S., Krisch L., Rohde E., Strunk D., Schallmoser K. (2021). Human Platelet Lysate for Good Manufacturing Practice-Compliant Cell Production. Int. J. Mol. Sci..

[19] Coppin L., Sokal E., Stephenne X. (2019). Thrombogenic Risk Induced by Intravascular Mesenchymal Stem Cell Therapy: Current Status and Future Perspectives. Cells.

[20] Tatsumi K., Ohashi K., Matsubara Y., Kohori A., Ohno T., Kakidachi H., Horii A., Kanegae K., Utoh R., Iwata T. (2013). Tissue factor triggers procoagulation in transplanted mesenchymal stem cells leading to thromboembolism. Biochem. Biophys. Res. Commun..

[21] Christy B.A., Herzig M.C., Montgomery R.K., Delavan C., Bynum J.A., Reddoch K.M., Cap A.P. (2017). Procoagulant activity of human mesenchymal stem cells. J. Trauma Acute Care Surg..

[22] Oeller M., Laner-Plamberger S., Hochmann S., Ketterl N., Feichtner M., Brachtl G., Hochreiter A., Scharler C., Bieler L., Romanelli P. (2018). Selection of Tissue Factor-Deficient Cell Transplants as a Novel Strategy for Improving Hemocompatibility of Human Bone Marrow Stromal Cells. Theranostics.

[23] Schallmoser K., Strunk D. (2009). Preparation of pooled human platelet lysate (pHPL) as an efficient supplement for animal serum-free human stem cell cultures. J. Vis. Exp..

[24] Schallmoser K., Bartmann C., Rohde E., Reinisch A., Kashofer K., Stadelmeyer E., Drexler C., Lanzer G., Linkesch W., Strunk D. (2007). Human platelet lysate can replace fetal bovine serum for clinical-scale expansion of functional mesenchymal stromal cells. Transfusion.

[25] Schallmoser K., Rohde E., Reinisch A., Bartmann C., Thaler D., Drexler C., Obenauf A.C., Lanzer G., Linkesch W., Strunk D. (2008). Rapid large-scale expansion of functional mesenchymal stem cells from unmanipulated bone marrow without animal serum. Tissue Eng. Part C Methods.

[26] Wildburger A., Payer M., Jakse N., Strunk D., Etchard-Liechtenstein N., Sauerbier S. (2014). Impact of autogenous concentrated bone marrow aspirate on bone regeneration after sinus floor augmentation with a bovine bone substitute--a split-mouth pilot study. Clin. Oral Implants Res..

[27] Grogan S.P., Barbero A., Winkelmann V., Rieser F., Fitzsimmons J.S., O’Driscoll S., Martin I., Mainil-Varlet P. (2006). Visual histological grading system for the evaluation of in vitro-generated neocartilage. Tissue Eng..

[28] Bartmann C., Rohde E., Schallmoser K., Purstner P., Lanzer G., Linkesch W., Strunk D. (2007). Two steps to functional mesenchymal stromal cells for clinical application. Transfusion.

[29] Prockop D.J., Gregory C.A., Spees J.L. (2003). One strategy for cell and gene therapy: Harnessing the power of adult stem cells to repair tissues. Proc. Natl. Acad. Sci. USA.

[30] Schallmoser K., Bartmann C., Rohde E., Bork S., Guelly C., Obenauf A.C., Reinisch A., Horn P., Ho A.D., Strunk D. (2010). Replicative senescence-associated gene expression changes in mesenchymal stromal cells are similar under different culture conditions. Haematologica.

[31] Dominici M., Le Blanc K., Mueller I., Slaper-Cortenbach I., Marini F., Krause D., Deans R., Keating A., Prockop D., Horwitz E. (2006). Minimal criteria for defining multipotent mesenchymal stromal cells. The International Society for Cellular Therapy position statement. Cytotherapy.

[32] Bieback K., Hecker A., Kocaomer A., Lannert H., Schallmoser K., Strunk D., Kluter H. (2009). Human alternatives to fetal bovine serum for the expansion of mesenchymal stromal cells from bone marrow. Stem Cells.

[33] Burnouf T., Strunk D., Koh M.B., Schallmoser K. (2016). Human platelet lysate: Replacing fetal bovine serum as a gold standard for human cell propagation?. Biomaterials.

[34] Gupta P., Hall G.N., Geris L., Luyten F.P., Papantoniou I. (2019). Human Platelet Lysate Improves Bone Forming Potential of Human Progenitor Cells Expanded in Microcarrier-Based Dynamic Culture. Stem Cells Transl. Med..

[35] Zayed M., Adair S., Dhar M. (2021). Effects of Normal Synovial Fluid and Interferon Gamma on Chondrogenic Capability and Immunomodulatory Potential Respectively on Equine Mesenchymal Stem Cells. Int. J. Mol. Sci..

[36] Alsaleh G., Sparsa L., Chatelus E., Ehlinger M., Gottenberg J.E., Wachsmann D., Sibilia J. (2010). Innate immunity triggers IL-32 expression by fibroblast-like synoviocytes in rheumatoid arthritis. Arthritis Res. Ther..

[37] Li Y.S., Luo W., Zhu S.A., Lei G.H. (2017). T Cells in Osteoarthritis: Alterations and Beyond. Front. Immunol..

[38] Liao G., Liao Y., Li D., Fu Z., Wu S., Cheng D., Ouyang Q., Tang Z., Zeng G., Liang X. (2021). Human Platelet Lysate Maintains Stemness of Umbilical Cord-Derived Mesenchymal Stromal Cells and Promote Lung Repair in Rat Bronchopulmonary Dysplasia. Front. Cell Dev. Biol..

[39] Bork S., Pfister S., Witt H., Horn P., Korn B., Ho A.D., Wagner W. (2010). DNA methylation pattern changes upon long-term culture and aging of human mesenchymal stromal cells. Aging Cell.

[40] Paz M.F., Fraga M.F., Avila S., Guo M., Pollan M., Herman J.G., Esteller M. (2003). A systematic profile of DNA methylation in human cancer cell lines. Cancer Res..

[41] Varley K.E., Gertz J., Bowling K.M., Parker S.L., Reddy T.E., Pauli-Behn F., Cross M.K., Williams B.A., Stamatoyannopoulos J.A., Crawford G.E. (2013). Dynamic DNA methylation across diverse human cell lines and tissues. Genome Res..

[42] Franzen J., Georgomanolis T., Selich A., Kuo C.C., Stoger R., Brant L., Mulabdic M.S., Fernandez-Rebollo E., Grezella C., Ostrowska A. (2021). DNA methylation changes during long-term in vitro cell culture are caused by epigenetic drift. Commun. Biol..

[43] Suelves M., Carrio E., Nunez-Alvarez Y., Peinado M.A. (2016). DNA methylation dynamics in cellular commitment and differentiation. Brief. Funct. Genom..

[44] Mead T.J., McCulloch D.R., Ho J.C., Du Y., Adams S.M., Birk D.E., Apte S.S. (2018). The metalloproteinase-proteoglycans ADAMTS7 and ADAMTS12 provide an innate, tendon-specific protective mechanism against heterotopic ossification. JCI Insight.

[45] Qi H., Jin M., Duan Y., Du X., Zhang Y., Ren F., Wang Y., Tian Q., Wang X., Wang Q. (2014). FGFR3 induces degradation of BMP type I receptor to regulate skeletal development. Biochim. Biophys. Acta.

[46] Harly C., Guillaume Y., Nedellec S., Peigne C.M., Monkkonen H., Monkkonen J., Li J., Kuball J., Adams E.J., Netzer S. (2012). Key implication of CD277/butyrophilin-3 (BTN3A) in cellular stress sensing by a major human gammadelta T-cell subset. Blood.

[47] Morimoto C., Schlossman S.F. (1998). The structure and function of CD26 in the T-cell immune response. Immunol. Rev..

[48] Strunk D., Egger C., Leitner G., Hanau D., Stingl G. (1997). A skin homing molecule defines the langerhans cell progenitor in human peripheral blood. J. Exp. Med..

[49] Maguer-Satta V., Besancon R., Bachelard-Cascales E. (2011). Concise review: Neutral endopeptidase (CD10): A multifaceted environment actor in stem cells, physiological mechanisms, and cancer. Stem Cells.

[50] Ding L., Vezzani B., Khan N., Su J., Xu L., Yan G., Liu Y., Li R., Gaur A., Diao Z. (2020). CD10 expression identifies a subset of human perivascular progenitor cells with high proliferation and calcification potentials. Stem Cells.

[51] Enyindah-Asonye G., Li Y., Ruth J.H., Spassov D.S., Hebron K.E., Zijlstra A., Moasser M.M., Wang B., Singer N.G., Cui H. (2017). CD318 is a ligand for CD6. Proc. Natl. Acad. Sci. USA.

[52] Liao H., Klaus C., Neumann H. (2020). Control of Innate Immunity by Sialic Acids in the Nervous Tissue. Int. J. Mol. Sci..

[53] Loh C.Y., Chai J.Y., Tang T.F., Wong W.F., Sethi G., Shanmugam M.K., Chong P.P., Looi C.Y. (2019). The E-Cadherin and N-Cadherin Switch in Epithelial-to-Mesenchymal Transition: Signaling, Therapeutic Implications, and Challenges. Cells.

[54] Lim J.Y., Kim T.W., Ryu D.B., Park S.S., Lee S.E., Kim B.S., Min C.K. (2021). Myeloma-Secreted Galectin-1 Potently Interacts with CD304 on Monocytic Myeloid-Derived Suppressor Cells. Cancer Immunol. Res..

[55] Su J., Guo L., Wu C. (2021). A mechanoresponsive PINCH-1-Notch2 interaction regulates smooth muscle differentiation of human placental mesenchymal stem cells. Stem Cells.

[56] Lopez-Garcia L., Castro-Manrreza M.E. (2021). TNF-alpha and IFN-gamma Participate in Improving the Immunoregulatory Capacity of Mesenchymal Stem/Stromal Cells: Importance of Cell-Cell Contact and Extracellular Vesicles. Int. J. Mol. Sci.

[57] Autenrieth S.E., Grimm S., Rittig S.M., Grunebach F., Gouttefangeas C., Buhring H.J. (2015). Profiling of primary peripheral blood- and monocyte-derived dendritic cells using monoclonal antibodies from the HLDA10 Workshop in Wollongong, Australia. Clin. Transl. Immunol..

[58] Kern S., Eichler H., Stoeve J., Kluter H., Bieback K. (2006). Comparative analysis of mesenchymal stem cells from bone marrow, umbilical cord blood, or adipose tissue. Stem Cells.

[59] Li H., Ghazanfari R., Zacharaki D., Ditzel N., Isern J., Ekblom M., Mendez-Ferrer S., Kassem M., Scheding S. (2014). Low/negative expression of PDGFR-alpha identifies the candidate primary mesenchymal stromal cells in adult human bone marrow. Stem Cell Rep..

[60] Hocking A.M. (2015). The Role of Chemokines in Mesenchymal Stem Cell Homing to Wounds. Adv. Wound Care.

[61] Thorp H., Kim K., Kondo M., Maak T., Grainger D.W., Okano T. (2021). Trends in Articular Cartilage Tissue Engineering: 3D Mesenchymal Stem Cell Sheets as Candidates for Engineered Hyaline-Like Cartilage. Cells.

[62] Moll G., Geissler S., Catar R., Ignatowicz L., Hoogduijn M.J., Strunk D., Bieback K., Ringden O. (2016). Cryopreserved or Fresh Mesenchymal Stromal Cells: Only a Matter of Taste or Key to Unleash the Full Clinical Potential of MSC Therapy?. Adv. Exp. Med. Biol..

[63] Reinisch A., Hernandez D.C., Schallmoser K., Majeti R. (2017). Generation and use of a humanized bone-marrow-ossicle niche for hematopoietic xenotransplantation into mice. Nat. Protoc..

[64] Pemmari A., Leppanen T., Hamalainen M., Moilanen T., Moilanen E. (2021). Chondrocytes from Osteoarthritis Patients Adopt Distinct Phenotypes in Response to Central TH1/TH2/TH17 Cytokines. Int. J. Mol. Sci.

[65] Lam J., Bellayr I.H., Marklein R.A., Bauer S.R., Puri R.K., Sung K.E. (2018). Functional Profiling of Chondrogenically Induced Multipotent Stromal Cell Aggregates Reveals Transcriptomic and Emergent Morphological Phenotypes Predictive of Differentiation Capacity. Stem Cells Transl. Med..

[66] Hoogduijn M.J., Lombardo E. (2019). Mesenchymal Stromal Cells Anno 2019: Dawn of the Therapeutic Era? Concise Review. Stem Cells Transl. Med..

[67] Galipeau J., Sensebe L. (2018). Mesenchymal Stromal Cells: Clinical Challenges and Therapeutic Opportunities. Cell Stem Cell.

[68] Moll G., Ankrum J.A., Kamhieh-Milz J., Bieback K., Ringden O., Volk H.D., Geissler S., Reinke P. (2019). Intravascular Mesenchymal Stromal/Stem Cell Therapy Product Diversification: Time for New Clinical Guidelines. Trends Mol. Med..

[69] Moll G., Ignatowicz L., Catar R., Luecht C., Sadeghi B., Hamad O., Jungebluth P., Dragun D., Schmidtchen A., Ringden O. (2015). Different Procoagulant Activity of Therapeutic Mesenchymal Stromal Cells Derived from Bone Marrow and Placental Decidua. Stem Cells Dev..

[70] Guiotto M., Raffoul W., Hart A.M., Riehle M.O., di Summa P.G. (2020). Human platelet lysate to substitute fetal bovine serum in hMSC expansion for translational applications: A systematic review. J. Transl. Med..

[71] Nguyen V.V.T., Witwer K.W., Verhaar M.C., Strunk D., van Balkom B.W.M. (2020). Functional assays to assess the therapeutic potential of extracellular vesicles. J. Extracell. Vesicles.

