# Supplementary material for: Batch Effects during Human Bone Marrow Stromal Cell Propagation Prevail Donor Variation and Culture Duration: Impact on Genotype, Phenotype and Function
Source: Cells. 2022 Mar 10;11(6):946. doi: 10.3390/cells11060946 (PMC8946746; doi:10.3390/cells11060946)
Supplement: Supplementary file 1 [file cells-11-00946-s001.zip › cells-1615419-supplementary.pdf]

Supplementary Table S1. List of genes in alphabetical order related to viral response, metabolism or interferon response found significantly upregulated in hPL compared to FBS.

Genes	Viral response	Metabolism	Interferon response	Gene Description
ABCC9	x			ATP-binding cassette subfamily C member 9
ACAA2		x		acetyl-CoA acyltransferase 2
ACADVL		x		acyl-CoA dehydrogenase very long chain
ACSL5		x		acyl-CoA synthetase long chain family member 5
ACSM5		x		acyl-CoA synthetase medium chain family member 5
ACSS1		x		acyl-CoA synthetase short chain family member 1
AKR1C2		x		aldo-keto reductase family 1 member C2
AKR1C3		x		aldo-keto reductase family 1 member C3
APOBEC3C	x			apolipoprotein B mRNA editing enzyme catalytic subunit 3C
APOBEC3D	x			apolipoprotein B mRNA editing enzyme catalytic subunit 3D
APOBEC3F	x			apolipoprotein B mRNA editing enzyme catalytic subunit 3F
APOBEC3G	x			apolipoprotein B mRNA editing enzyme catalytic subunit 3G
ARRB1	x			arrestin beta 1
ASPA		x		aspartoacylase
BST2	x		x	bone marrow stromal cell antigen 2
C19orf66	x		x	
C4A	x			complement C4A (Rodgers blood group)
C4B	x			complement C4B (Chido blood group)
CARD16	x			caspase recruitment domain family member 16
CASP1	x		x	caspase 1
CASP7	x			caspase 7
CASP8	x			caspase 8
CCL2			x	C-C motif chemokine ligand 2
CD40	x		x	CD40 molecule
CD74	x	x		CD74 molecule
CDO1		x		cysteine dioxygenase type 1
CLU	x			clusterin
CPT1A		x		carnitine palmitoyltransferase 1A
DDX58	x			DExD/H-box helicase 58
DDX60	x			DExD/H-box helicase 60
DECR1		x		2,4-dienoyl-CoA reductase 1
DLD		x		dihydrolipoamide dehydrogenase
DTX3L	x			deltex E3 ubiquitin ligase 3L
ECH1		x		enoyl-CoA hydratase 1
ECI2		x		enoyl-CoA delta isomerase 2

EPHX2		x		epoxide hydrolase 2
ETFA		x		electron transfer flavoprotein subunit alpha
ETFB		x		electron transfer flavoprotein subunit beta
F2R	x			coagulation factor II thrombin receptor
F2RL1	x			F2R like trypsin receptor 1
FAH		x		fumarylacetoacetate hydrolase
FMC1		x		formation of mitochondrial complex V assembly factor 1 homolog
GALM		x		galactose mutarotase
GBP2			x	guanylate binding protein 2
GSTZ1		x		glutathione S-transferase zeta 1
HADHA		x		hydroxyacyl-CoA dehydrogenase trifunctional multienzyme complex subunit alpha
HADHB		x		hydroxyacyl-CoA dehydrogenase trifunctional multienzyme complex subunit beta
HLA-DPA1			x	major histocompatibility complex, class II, DP alpha 1
HLA-DPB1			x	major histocompatibility complex, class II, DP beta 1
IFI16	x			interferon gamma inducible protein 16
IFI30			x	IFI30 lysosomal thiol reductase
IFI6	x			interferon alpha inducible protein 6
IFIH1	x			interferon induced with helicase C domain 1
IFIT3	x			interferon induced protein with tetratricopeptide repeats 3
IFITM2	x		x	interferon induced transmembrane protein 2
IFT57	x			intraflagellar transport 57
IL15	x			interleukin 15
IRF1	x		x	interferon regulatory factor 1
IRF2	x		x	interferon regulatory factor 2
KCNJ8	x			potassium inwardly rectifying channel subfamily J member 8
LXN	x			latexin
MX1	x			MX dynamin like GTPase 1
MX2	x			MX dynamin like GTPase 2
MYC	x			MYC proto-oncogene, bHLH transcription factor
NELFCD	x			negative elongation factor complex member C/D
NMI			x	N-myc and STAT interactor
NTSR1		x		neurotensin receptor 1
NUDT7		x		nudix hydrolase 7
OAS3	x		x	2′-5′-oligoadenylate synthetase 3
OASL	x		x	2′-5′-oligoadenylate synthetase like
PCCA		x		propionyl-CoA carboxylase subunit alpha
PDK4		x		pyruvate dehydrogenase kinase 4
PLCD4		x		phospholipase C delta 4
PLSCR1	x			phospholipid scramblase 1
PML	x		x	PML nuclear body scaffold
PPARG	x	x	x	peroxisome proliferator-activated receptor gamma
PPIB	x			peptidylprolyl isomerase B

PSMB8	x			proteasome 20S subunit beta 8
PSMB9	x			proteasome 20S subunit beta 9
PTGS1		x		prostaglandin-endoperoxide synthase 1
PYCARD	x			PYD and CARD domain containing
RARRES3		x		retinoic acid receptor responder protein 3
RTP4	x			receptor transporter protein 4
SERPINF1	x			serpin family F member 1
SOCS3			x	suppressor of cytokine signaling 3
TKFC	x	x		triokinase and FMN cyclase
TLR3	x		x	toll-like receptor 3
TNFSF10	x			TNF superfamily member 10
TRIM21	x		x	tripartite motif-containing 21
TSPAN32	x			tetraspanin 32
TSPAN6	x			tetraspanin 6
